# A New Understanding of TMEM119 as a Marker of Microglia

**DOI:** 10.3389/fncel.2022.902372

**Published:** 2022-06-13

**Authors:** Chunsheng Ruan, Wassim Elyaman

**Affiliations:** Department of Neurology, Columbia University Medical Center, New York, NY, United States

**Keywords:** microglia, marker, TMEM119, intracellular domain, extracellular domain

Neuroinflammation is a hallmark of many neurological diseases, including traumatic brain injury (TBI), which is usually characterized by two pathological hallmarks: activation of microglia and infiltration of blood-borne macrophages (Hanisch and Kettenmann, 2007; Ransohoff and Perry, 2009; Zanier et al., 2015). Until recently, the two cell populations were antigenically indistinguishable due to a shared myeloid lineage (Varol et al., 2015; Prinz et al., 2017), which complicates our understanding of their neuroinflammatory response in the brain. Recently, advanced single-cell RNA sequencing profiling technologies have uncovered several protein markers of microglia, including transmembrane protein 119 (TMEM119) (Bennett et al., 2016). Under physiological conditions, TMEM119 is specifically expressed in homeostatic human and murine microglia, but not in other brain-resident cells nor in infiltrating macrophages (Bennett et al., 2016; Satoh et al., 2016; Ruan et al., 2020), which enables the differentiation between brain-resident microglia and infiltrating blood-derived macrophages. More recent studies, including the present one by Mercurio and colleagues, have tested its efficacy in reactive microglia, and their findings challenge the use of TMEM119 as a robust marker of microglia, especially under pathological conditions.

Mercurio et al. (2022) reported an upregulation of *Tmem119* gene expression in frozen brain sections of TBI (Day 7) by RT-PCR and using *in situ* hybridization experiments; they further confirmed its upregulation, specifically in the contused area and surrounding regions, along with increased microglial activation. These findings are consistent with a study showing the increased *Tmem119* mRNA level in frozen tissue of the frontal cortex from patients with Alzheimer’s (Satoh et al., 2016), which suggests that increased *Tmem119* gene expression in the whole population of microglia is paralleled with tissue inflammation. However, after isolation by fluorescence-activated cell sorting (FACS), Li and colleagues found a reduced *Tmem119* mRNA level in CD11b^+^CD45*^Int^* microglia (bulk population) on Day 1 post-intracerebral hemorrhage (Li et al., 2019). In line with this finding, transcriptome studies using the RNA-seq technique also showed a reduced *Tmem119* gene expression in isolated reactive microglia (Keren-Shaul et al., 2017; Krasemann et al., 2017; Masuda et al., 2020). Interestingly, transforming growth factor beta (TGF-β) induced an upregulated *Tmem119* gene expression in cultured mouse microglia (Attaai et al., 2018), while lipopolysaccharide (LPS), interleukin 4 (IL-4), or interferon gamma (IFN-γ) induced a downregulated *Tmem119* gene expression in cultured human microglia (Bennett et al., 2016; Satoh et al., 2016; Van Wageningen et al., 2019), although Satoh et al. (2016) also found no change of *Tmem119* gene expression in human microglia HMO6 cells after treatment of LPS or TGF-β. Taken together, although the gene regulation of *Tmem119* in reactive and diseased microglia needs further clarification, it is most likely that the different patterns of *Tmem119* gene expression in inflamed tissues compared to isolated microglial cells are due to the presence of inflammatory milieu and/or other microenvironmental stimuli. Single-cell RNA-seq data should be helpful to characterize different subsets of microglia with differential levels of *Tmem119* gene expression in health and disease.

Apart from the regulation of gene expression, Mercurio et al. (2022) found a significant reduction in the TMEM119 protein expression level in the injured cortex on Day 4 after TBI by Western blot, and on Days 4 and 7 after TBI by immunostaining (along with an increased Iba1 immunoreactivity), which suggests a downregulation of TMEM119 in reactive microglia. This finding is consistent with all the current findings so far regarding the protein expression of TMEM119 in the reactive microglia (Satoh et al., 2019; Van Wageningen et al., 2019; Cao et al., 2021; Marzan et al., 2021; Young et al., 2021). However, it is rare to see a complete loss of TMEM119 immunoreactivity in Iba1-positive myeloid cells (Li et al., 2019; Young et al., 2021), and only a proportion not all of the reactive microglia showed lower expression of TMEM119 (Kenkhuis et al., 2022). This suggests that TMEM119 protein is likely still involved in the activation of microglia, although the process of its downregulation in reactive microglia remains unclear. The present data in TBI (Mercurio et al., 2022) are in agreement with the findings in the brain of patients with Alzheimer’s disease, in which the TMEM119 immunoreactivity is greatly reduced in the amyloid plaque-surrounding microglia and is often associated with a significant increase in Iba1 reactivity (Swanson et al., 2020; Kenkhuis et al., 2022; Vankriekelsvenne et al., 2022). Moreover, the downregulation of TMEM119 protein in the contused area is in line with the findings at the sites of neurodegeneration where TMEM119 immunoreactivity is reduced in the disease-associated microglia (Krasemann et al., 2017; Deczkowska et al., 2018; Satoh et al., 2019). In these microglia, the expression of TMEM119 is not only reduced but is also redistributed to the phagosomes and/or the processes (Young et al., 2021; Kenkhuis et al., 2022), suggesting that plasma membrane TMEM119 may be involved in the phagocytic function of microglia, such as apoptotic cells, cell debris or abnormal protein aggregates (i.e., amyloid plaques in Alzheimer’s). It is known that, during phagocytosis, the plasma membrane is embedded to form phagosomes that are transported to fuse with acidic lysosomes for non-specific degradation (Underhill and Goodridge, 2012). This suggests that the observed redistribution of TMEM119 to the phagosomes and the phagosome-lysosome degradation pathway may result in the downregulation of TMEM119 protein in reactive microglia. However, this hypothesis can not explain the data in our previous study (Ruan et al., 2020), in which the brain cortex of Tmem119-tdTomato reporter mice that were exposed to laser injury showed robust activation of Tmem119-tdTomato positive microglia. If the TMEM119 protein is reduced during activation by the theory proposed above, we should see a diminished tdTomato fluorescence at the injury site over time. Instead, we were able to detect a strong TMEM119 signal (shown in red) in reactive microglia. Our data suggest that there is, potentially, an alternative pathway other than non-specific degradation involving the regulation of the TMEM119 protein level during microglial activation.

Given that TMEM119 is a type-I transmembrane protein structurally similar to well-studied amyloid precursor protein (APP) and Notch proteins, it is very likely that a full length (FL) of TMEM119 can also be cleaved into an extracellular domain (ECD) and intracellular domain (ICD) fragments. This hypothesis can be confirmed using the anti-TMEM119 (extracellular) antibody (#ANR-175) by Western blot. This antibody hypothetically should label both the full-length protein and the ECD fragment (as shown in Figure 1A). The reason why TMEM119-ECD is not detected in mouse BV-2 microglial cell lysate is that we believe that ECD has been released into culture media (Figure 1B). Based on a smaller size of TMEM119 detected in the small extracellular vesicles in Figure 3A (right panel; Brenna et al., 2020) using anti-TMEM119 (intracellular) antibody (#66948-1-Ig), the band corresponds to the TMEM119-ICD. With this in mind, it seems obvious to conclude that the red fluorescence in Figure 1C labels a combination of TMEM119-FL-tdTomato and TMEM119-ICD-tdTomato positive microglia (based on the vector design of the Tmem119-tdTomato mice, tdTomato is linked to the ICD). Considering the significant decrease of TMEM119 in reactive microglia, theoretically, the microglia at the lesion site should mostly be TMEM119-ICD-tdTomato positive. Taken together, our data suggest that the intracellular domain of TMEM119 instead of its full length could be an alternative marker for microglia, and, by some means, the secreted extracellular domain of TMEM119 could also be useful as a biomarker of reactive microglia; however, further studies are required. Considering our hypothesis above, the upregulation of *Tmem119* gene expression in reactive microglia (if it is true) is most likely a compensatory pathway. On the contrary, if reactive microglia, indeed, show downregulation of *Tmem119* gene expression, then this downregulation is likely a negative regulation leading to less TMEM119 expression on the plasma membrane. Given that two studies mentioned earlier have shown an overall upregulation of *Tmem119* gene expression in microglia (in both homeostatic and active forms) in the injured tissues (Satoh et al., 2016; Mercurio et al., 2022), then the homeostatic microglia close to the injury should have an upregulation of *Tmem119* gene expression, which needs to be further examined.

**FIGURE 1:**
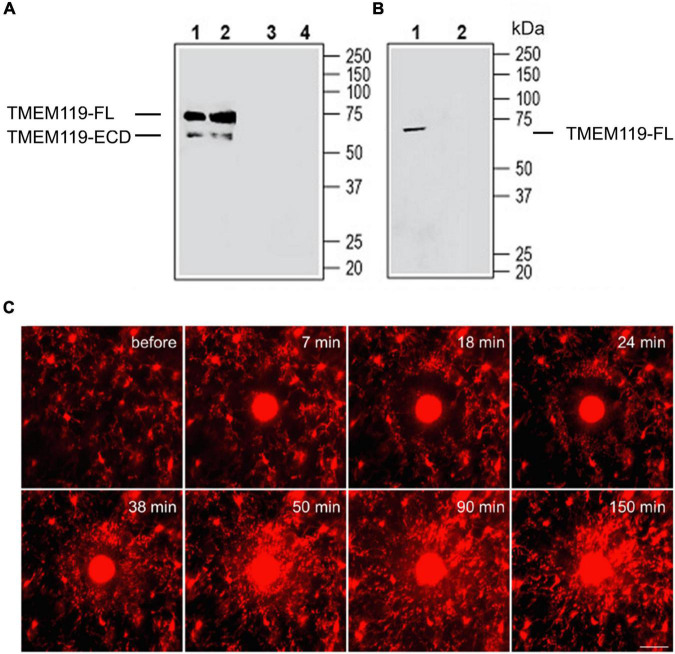
Validation of anti-TMEM119 antibodies by Western blot and two-photon live imaging of TMEM119-tdTomato^+^ microglia *in vivo* after laser-mediated injury. **(A)** Western blot analysis of mouse brain membranes (Lanes 1 and 3) and rat brain membranes (Lanes 2 and 4): 1, 2. Anti-TMEM119 (extracellular) antibody (#ANR-175), (1:200); 3, 4. An anti-TMEM119 (extracellular) antibody, preincubated with TMEM119 (extracellular) blocking peptide (#BLP-NR175). **(B)** Western blot analysis of mouse BV-2 microglia cell lysate: 1. An anti-TMEM119 (extracellular) antibody (#ANR-175), (1:200); 2. An anti-TMEM119 (extracellular) antibody, preincubated with TMEM119 (extracellular) blocking peptide (#BLP-NR175). FL, full length; ECD, extracellular domain. Data are cited from https://www.alomone.com/p/anti-tmem119-extracellular-antibody/ANR-175 with permission of the company. **(C)** After localized ablation inside the primary sensory cortex, neighboring TMEM119-tdTomato^+^ microglia respond quickly with extended processes and bulbous termini. Time-lapse images show that microglia formed a spherical containment around the laser lesion site. Scale bar, 30 μm. The red color indicates the mix of TMEM119-FL-tdTomato and TMEM119-ICD-tdTomato microglia. FL, full length; ICD, intracellular domain. Data are cited from Ruan et al. (2020) Brain Behav Immun.

To conclude, the present commentary discusses the current findings of TMEM119 as a marker for human and murine microglia under physiological and pathological conditions and proposes a novel hypothesis regarding TMEM119 regulation in reactive microglia. By limited data so far, it is suggested that the downregulation of TMEM119 in reactive microglia is most likely an outcome of both the non-specific phagosome-lysosome degradation pathway and the specific proteolytic cleavage pathway. Detection of the extracellular domain of TMEM119 in cerebrospinal fluid or blood by ELISA could be an easy way to monitor the activation state of microglia in patients and animal models of brain diseases. Furthermore, pathological detection of the intracellular domain of TMEM119 in fixed brain tissues could be a better marker than the full-length TMEM119 to label the whole population of microglia, which may enable the differentiation of CNS-resident microglia (including reactive microglia) and blood-infiltrating macrophages. Western blot analysis targeting the extracellular or intracellular domain of TMEM119 could also be informatic for the activation of microglia. In addition, our Tmem119-tdTomato reporter mice could be another useful tool to track all microglia phenotypes (both homeostatic and reactive microglia) *in vivo* by live imaging. However, since TMEM119 is majorly cleaved or degraded during the activation of microglia, its application as a surface marker for flow cytometry analysis of microglia is weakened, and a combination with other relevant markers is recommended. Overall, TMEM119 or its fragments are, indeed, constantly expressed in the whole population of microglia, which suggests that TMEM119 is still a promising microglia marker.

## Author Contributions

CR and WE contributed ideas and wrote the final manuscript. Both authors contributed to the article and approved the submitted version.

## Conflict of Interest

The authors declare that the research was conducted in the absence of any commercial or financial relationships that could be construed as a potential conflict of interest.

## Publisher’s Note

All claims expressed in this article are solely those of the authors and do not necessarily represent those of their affiliated organizations, or those of the publisher, the editors and the reviewers. Any product that may be evaluated in this article, or claim that may be made by its manufacturer, is not guaranteed or endorsed by the publisher.
